# Supplementary material for: The causal role of circulating inflammatory markers in osteoporosis: a bidirectional Mendelian randomized study
Source: Front Immunol. 2024 Jul 18;15:1412298. doi: 10.3389/fimmu.2024.1412298 (PMC11291241; doi:10.3389/fimmu.2024.1412298)
Supplement: Supplementary file 1 [file DataSheet_1.zip › supplementary material/CIM-OSTEOPOROSIS /GCST90274788.csv_forest.pdf]

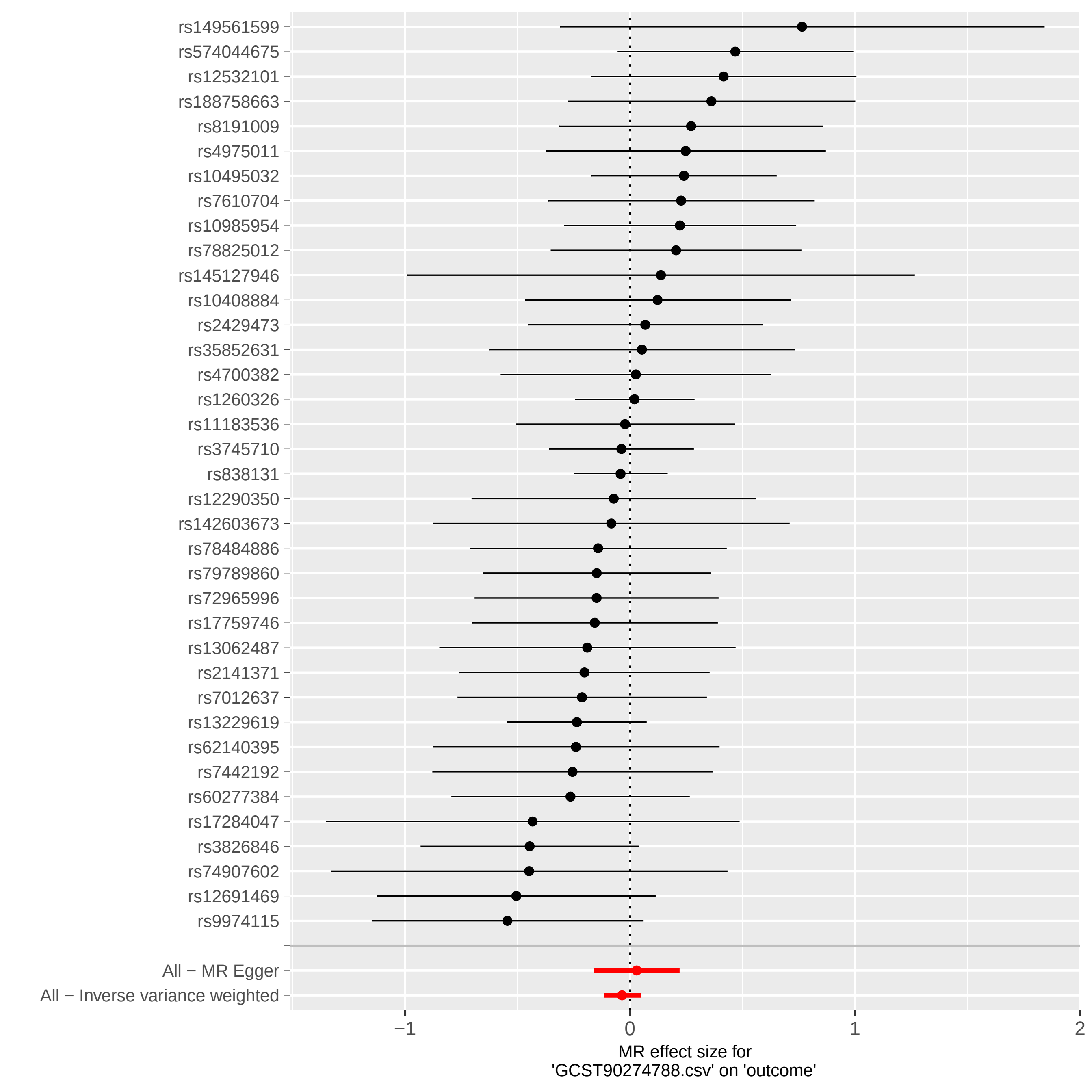
rs149561599
rs574044675
rs12532101
rs188758663
rs8191009
rs4975011
rs10495032
rs7610704
rs10985954
rs78825012
rs145127946
rs10408884
rs2429473
rs35852631
rs4700382
rs1260326
rs11183536
rs3745710
rs838131
rs12290350
rs142603673
rs78484886
rs79789860
rs72965996
rs17759746
rs13062487
rs2141371
rs7012637
rs13229619
rs62140395
rs7442192
rs60277384
rs17284047
rs3826846
rs74907602
rs12691469
rs9974115
All − MR Egger
All − Inverse variance weighted
−1
0
1
2
MR effect size for
'GCST90274788.csv' on 'outcome'
